# Breaking through Multiple Myeloma: A Paradigm for a Comprehensive Tumor Ecosystem Targeting

**DOI:** 10.3390/biomedicines11072087

**Published:** 2023-07-24

**Authors:** Antonio G. Solimando, Markus Krebs, Vanessa Desantis, Donatello Marziliano, Ingrid Catalina Caradonna, Arcangelo Morizio, Antonella Argentiero, Endrit Shahini, Max Bittrich

**Affiliations:** 1 Unit of Internal Medicine and Clinical Oncology “G. Baccelli”, Department of Precision and Regenerative Medicine and Ionian Area, University of Bari Aldo Moro Medical School, 70124 Bari, Italy; d.marziliano1@studenti.uniba.it; 2 Comprehensive Cancer Center Mainfranken, University Hospital Würzburg, 97080 Würzburg, Germany; krebs_m@ukw.de; 3 Department of Urology and Pediatric Urology, University Hospital Würzburg, 97080 Würzburg, Germany; 4 Department of Precision and Regenerative Medicine and Ionian Area, Pharmacology Section, University of Bari Aldo Moro Medical School, 70124 Bari, Italy; vanessa86.desantis@gmail.com (V.D.); i.caradonna1@studenti.uniba.it (I.C.C.); 5 Orthopedics and Traumatology Unit ASL BA-Ospedale della Murgia “Fabio Perinei”, 70022 Altamura, Italy; 6 IRCCS Istituto Tumori “Giovanni Paolo II” of Bari, 70124 Bari, Italy; 7 Gastroenterology Unit, National Institute of Gastroenterology—IRCCS “Saverio de Bellis”, 70013 Castellana Grotte, Italy; 8 Department of Internal Medicine II, University Hospital Würzburg, 97080 Würzburg, Germany

**Keywords:** multiple myeloma, microenvironment, immunotherapy, monoclonal antibody

## Abstract

Multiple myeloma (MM) is a cancerous condition characterized by the proliferation of plasma cells within the hematopoietic marrow, resulting in multiple osteolytic lesions. MM patients typically experience bone pain, kidney damage, fatigue due to anemia, and infections. Historically, MM was an incurable disease with a life expectancy of around three years after diagnosis. However, over the past two decades, the development of novel therapeutics has significantly improved patient outcomes, including response to treatment, remission duration, quality of life, and overall survival. These advancements include thalidomide and its derivatives, lenalidomide and pomalidomide, which exhibit diverse mechanisms of action against the plasma cell clone. Additionally, proteasome inhibitors such as bortezomib, ixazomib, and carfilzomib disrupt protein degradation, proving specifically toxic to cancerous plasma cells. Recent advancements also involve monoclonal antibodies targeting surface antigens, such as elotuzumab (anti-CS1) and daratumumab (anti-CD38), bispecific t-cell engagers such as teclistamab (anti-BCMA/CD3) and Chimeric antigen receptor T (CAR-T)-based strategies, with a growing focus on drugs that exhibit increasingly targeted action against neoplastic plasma cells and relevant effects on the tumor microenvironment.

## 1. Introduction

Multiple myeloma (MM) is a neoplastic disease originating from monoclonal plasma cells that proliferate and expand at the level of the hematopoietic marrow, causing damage to several organs. Monoclonal plasma cells produce, in most cases, the so-called monoclonal component, consisting of identical immunoglobulins that migrate homogeneously to the electrophoretic protein separation and thus form the typical monoclonal peak.

MM accounts for about 1–2% of all malignancies and about 10% of hematologic malignancies [1,2]; its incidence in the United States of America is equal to 6.6 cases per 100,000 inhabitants. MM typically affects the elderly, with a median age at diagnosis of about 70 years; about 30% of patients are over 75 years old at diagnosis and less than 10% are between 20 and 40 years old. The causes of MM onset are still largely unknown. It is possible that genetic predisposing factors and their interaction with the environment play a role in the development of the disease. One known etiological agent is exposure to ionizing radiation [3]. Another risk factor is exposure to pesticides, heavy metals, fine dust, nano compounds, cigarette smoke, and alcohol. Finally, familial cases are described in the literature.

In most patients, the onset of the disease in its symptomatic form (active or symptomatic MM) is preceded by a phase of “monoclonal gammopathy of uncertain significance” (MGUS) and by a phase of “indolent multiple myeloma” or of “smoldering” type. These phases are both asymptomatic and therefore often not clinically evident.

Myeloma results from a neoplastic transformation that occurs at the level of the B lymphocyte lineage. Genetic modifications and interactions with the marrow microenvironment are responsible for neoplastic proliferation. The neoplastic transformation occurs at the level of B cells of the post-germinative center, i.e., in the last stages of B cell maturation and differentiation, most likely involving a memory B cell or a plasmablast.

The genetic alterations implicated in the pathogenesis of myeloma are complex. Primary and secondary genetic abnormalities (predominantly translocations) have been recognized. Primary translocations involve the 14q32 heavy chain (IgH) region of immunoglobulins in 40–50% of patients and are common to myeloma and monoclonal gammopathies of uncertain significance [4]. These lesions are therefore indispensable for the development of gammopathy, while a second event (“second hit”) is required for neoplastic evolution. Secondary lesions then appear with disease progression, and include loss of chromosome 13, activating mutations of NRAS, KRAS, and MAPK oncogenes, inactivating mutations or deletions of p53, and inactivation of PTEN [5,6,7,8].

Patients with MM can be divided into two main groups: those with hyperdiploid and those with non-hyperdiploid plasma cell clones, according to the chromosomal makeup of the monoclonal plasma cells. Based on the hyper- or hypo-diploidy and chromosomal translocations involving the 14q32 region, different subgroups of patients with different prognoses can be identified [9,10,11]. The chromosomal/genomic characteristics of the patients can therefore be used as prognostic parameters: translocations t(4;14), t(14;16), or deletion of the short arm of both chromosome 17 and 1 together with gain 1q21 are correlated with a reduced survival. Currently, the chromosomal characterization of the disease is not yet sufficient to correspond to specific standardized therapeutic approaches, although new evidence will likely enable therapy stratification based on specific mutations.

The process of linear tumorigenesis, characterized by the progressive acquisition of different mutations that confer a selective advantage to the neoplastic clone, is questioned by genetic studies conducted on monoclonal plasma cells in different phases of the disease. In fact, according to the recent theory of clonal evolution, many clones of genotypically different plasma cells coexist within the same patient, and it is their ratio and balance determining the natural history of the disease [12].

**Figure 1:**
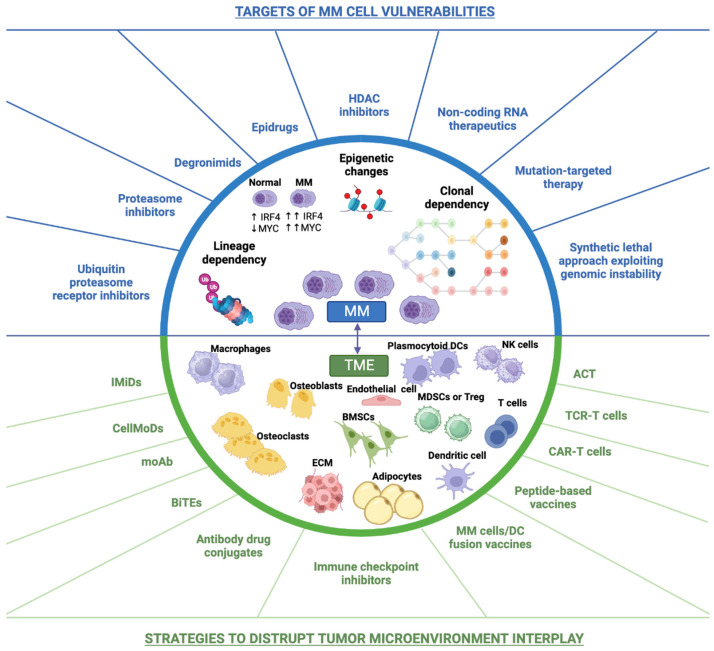
Significant advancements have been made in therapeutic approaches for multiple myeloma. These approaches can be broadly categorized into two main strategies: those targeting the toxicity of multiple myeloma cells (represented by the color blue) and those aiming to disrupt the interplay between MM cells and the tumor microenvironment (represented by the color green). MM = Multiple myeloma; TME = Tumor microenvironment; HDAC = Hystone deacetylase; IRF4 = Interferon regulatory factor 4; IMiDs = Immunomodulatory drugs; ACT = adoptive T cells; TCR = T cell receptor; NK = Natural killer; CAR-T = Chimeric antigen receptor T; DC = Dendritic cells; MDSC = Myeloid derived suppressor cells; Treg = T regulatory cells; BMSC = Bone marrow stromal cells; ECM = Extracellular matrix; moAb = Monoclonal antibody; CellMoDs = Cereblon E3 ligase modulators; BiTEs = Bispecific T cell engager. Figure created by BioRender, publication license n. CT25JR89XW.

The bone marrow microenvironment is essential for the development of monoclonal plasma cells [13,14]. The adhesion of plasma cells to hematopoietic cells induces the secretion of cytokines and growth factors (interleukin-6, vascular endothelial growth factor, insulin-like growth factor 1, IGF-1). The cell adhesion system hijacks the hematopoietic homeostasis [15,16,17,18,19] with the creation of autocrine and paracrine circuits, which support plasma cell growth. Furthermore, the adhesion of plasma cells to extracellular matrix proteins induces the production of proteins that regulate the cell cycle, and of anti-apoptotic proteins [20,21,22] (Figure 1).

**Figure 2:**
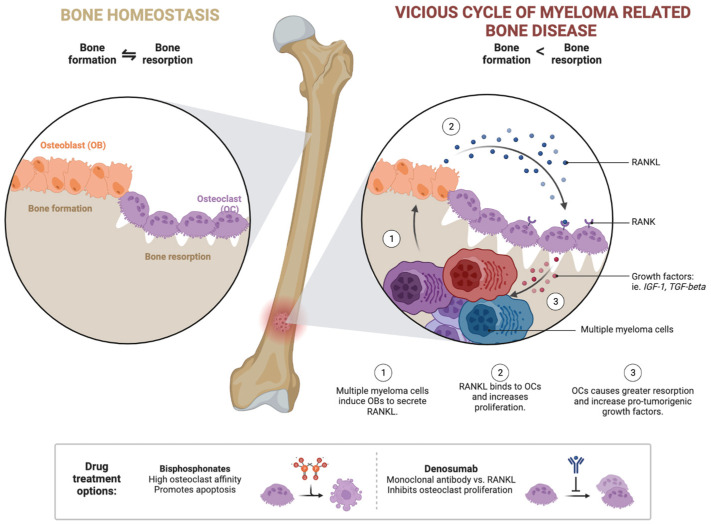
This figure illustrates the pathomechanisms of multiple myeloma (MM) bone disease characterized by increased osteoclast activity and decreased osteoblast function and resulting in bone destruction and skeletal complications. The figure highlights key factors that contribute to MM bone disease, including: (i) increased osteoclast activity: MM cells produce factors, such as receptor activator of nuclear factor kappa-B ligand (RANKL), that activate osteoclasts and promote bone resorption. This leads to an increase in bone turnover and the release of factors that further stimulate MM cell growth. (ii) Decreased osteoblast function: MM cells and their microenvironment produce factors, such as Dickkopf-1 (DKK1) and sclerostin that inhibit osteoblast differentiation and function, impairing bone formation and repair. (iii) Disruption of bone remodeling: The dysregulation of osteoclast and osteoblast activity in MM leads to an imbalance in bone remodeling, resulting in the accumulation of abnormal bone tissue and the development of lytic lesions, fractures, and bone pain. (iv) immune dysregulation: The immune dysregulation in MM can also contribute to bone disease by promoting osteoclast activation and inhibiting osteoblast function. For example, activated T cells and cytokines, such as interleukin-6 (IL-6), can stimulate osteoclast activity and inhibit osteoblast differentiation. Understanding the pathomechanisms of MM bone disease is crucial for the development of effective therapeutic strategies to prevent and treat skeletal complications. Targeting osteoclast activity, promoting osteoblast function, and restoring immune regulation are promising approaches for the treatment of MM bone disease. Figure created by BioRender, publication license n. QH258ANAV7.

The osteolytic lesions typical of myeloma are the product of an imbalance between bone production by osteoblasts and bone destruction by osteoclasts. The increase in osteoclast activity observable in patients with MM is due to an imbalance between the receptor activator of nuclear factor kB (RANK) and osteoprotegerin (OPG), caused, in turn, by the increased production of RANK ligand (RANKL) and the decreased production of osteoprotegerin. Stromal damage is so severe that bone reconstruction is rarely observed, even in patients in complete remission (Figure 2) [17,23].

The clinical picture of MM is characterized by several symptoms, accounting for the expression of the organ damage determined by both the proliferation of plasma cells, their interaction with the surrounding immune microenvironment, as in other solid and hematological malignancies [24,25], and by the production of whole and fractionated immunoglobulins [26,27,28] (Figure 2). The characteristic symptoms of MM include bone pain related to pathological bone abnormalities, kidney damage related to the increased production of immunoglobulins or their fractions, marked asthenia due to anemia, and infections [29].

Recent advances in our understanding of the MM tumor ecosystem have revealed the complex interplay between tumor cells, immune cells, stromal cells, and extracellular matrix components in driving disease development and resistance to therapy.

In response, a paradigm shift towards a comprehensive tumor ecosystem targeting approach has emerged, with the goal of targeting multiple components of the MM microenvironment to improve patient outcomes. This approach encompasses a range of therapeutic strategies, including immunomodulatory drugs, monoclonal antibodies, cell-based therapies, and targeted therapies, that aim to disrupt the interplay between tumor cells and their microenvironment.

In this article, we will review the current understanding of the MM tumor ecosystem and the rationale for a comprehensive targeting approach. We will discuss the key components of the MM tumor ecosystem, including immune cells, stromal cells, and extracellular matrix components, and the molecular mechanisms that underlie their interactions with tumor cells. We will also highlight the promising therapeutic strategies that are being developed to target the MM tumor ecosystem and improve patient outcomes [11,30].

### Overview on Multiple Myeloma Impact

MM is currently considered as a treatable, but incurable, disease. However, the outlook for life and care of the MM patient has changed dramatically over the past two decades. In fact, until the end of the last millennium, myeloma therapy was based on the use of conventional chemotherapy, with different intensities in young patients eligible for autologous stem cell transplantation, as opposed to elderly patients, for whom melphalan and cortisone were the standard treatment [31,32]. The survival of these patients averaged no more than 3–5 years. By contrast, in the last two decades, the introduction of modern drugs and of different treatment strategies has profoundly increased the quality of response to therapies, the duration of remission, the quality of life, and ultimately the survival of patients affected by MM.

New drugs include thalidomide and its second- and third-generation analogs, lenalidomide and pomalidomide. These drugs act on the plasma cell clone through various mechanisms of action, including direct cytotoxicity, anti-angiogenic effects, protein folding and production in plasma cells, and anti-tumor immunity. In addition, proteasome inhibitors, such as bortezomib, ixazomib, and carfilzomib, interrupt the degradation of proteins and are specifically cytotoxic for neoplastic plasma cells. Recently, monoclonal antibodies directed against surface antigens, such as daratumumab (anti-CD38), have been tested and finally approved in the treatment of myeloma.

Moreover, elotuzumab (anti-CS1) is indicated in combination with pomalidomide and dexamethasone for the treatment of adult patients with relapsed and refractory multiple myeloma who have received at least two prior therapies including lenalidomide and a proteasome inhibitor demonstrating disease progression on the last therapy.

Significant attention is being paid to drugs with an increasingly selective action against neoplastic plasma cells.

## 2. New Insights into the Biology of the Disease

Almost all MM cases are preceded by two clinically silent phases, that of MGUS and its evolution into smoldering MM (asymptomatic, SMM), which finally results in the symptomatic form of MM, characterized by the appearance of organ damage related to the proliferation of myeloma cells or their production of monoclonal paraprotein [33,34]. The fact that many patients receive a diagnosis of MM without a previous finding of MGUS or SMM is due to the absence of any clinical signs or symptoms.

The incidence of MGUS in the general population is approximately 3%, with an evolution rate to symptomatic MM that remains constant over time, at approximately 1% per year. On the contrary, the probability of evolution from asymptomatic to symptomatic myeloma decreases over time, being equal to 5% per year in the first 5 years after diagnosis, but then decreasing to 3% in the following 5 years, and dropping to 1.5% after 10 years [35].

MGUS is defined as the presence of a serum monoclonal component of less than 3 g/dl or in urine (Bence-Jones proteinuria) of less than 0.5 g/day, in association with a percentage of bone marrow monoclonal plasma cells of less than 10%.

The diagnosis of MM, on the other hand, is based on evidence of at least 10% monoclonal plasma cells in the bone marrow. The presence or absence of signs or symptoms evocative of organ damage related to the proliferation of marrow plasma cells is the discriminant factor defining asymptomatic or symptomatic MM.

SMM (asymptomatic or indolent) is a clinical picture characterized by the presence of at least 10% monoclonal plasma cells in the marrow (or a monoclonal component in serum > 3 g/dl or urine > 500 mg/day) in the absence of signs or symptoms of organ damage related to proliferative disease. Biologically, SMM is a heterogeneous condition; in fact, it can present features similar those described in cases of MGUS, with a truly indolent clinical course. In other cases, it can present characteristics closer to those observed in patients with symptomatic MM, who have an increased risk of clinical progression. This clinical entity is therefore distinguished from MGUS pictures first by the risk of progression to MM; in the first case, this risk is equal to 10% per year, whereas in the second it is equal to 1% per year. In a study conducted in the Swedish Myeloma Registry, 14% of patients diagnosed with MM were classified as SMM [36].

Until the end of 2014, the diagnosis of symptomatic MM therefore required the presence of at least 10% of bone marrow monoclonal plasma cells (or a serum monoclonal component ≥ 3 g/dl), accompanied by the presence of at least one of the signs or symptoms of damage related to myeloma and commonly represented (and summarized) by the CRAB acronym: hypercalcemia, renal failure, anemia, and bone lesions. In November 2014, however, the International Myeloma Working Group (IMWG) released new guidelines regarding the new diagnostic criteria for myeloma. Two substantial innovations were introduced: a better definition of the CRAB criteria and the introduction of three additional factors that contribute to define MM as an active disease [17,35,37].

As regards the clarification of the CRAB criteria, the definition of renal failure has been refined and, alongside a creatinine level >2 mg/dl, a creatinine clearance value <40/mL has been added. In addition, in the evaluation of bone lesions, computed tomography (CT) and positron emission tomography (PET-CT) have been added to conventional radiography.

As regards the symptomatic criteria of myeloma, the IMWG has introduced, alongside the well-known CRAB criteria, three new myeloma-defining events (MDE), i.e., those clinical-laboratory elements in the presence of which myeloma can be defined as symptomatic and therefore warranting treatment. These include taking into account a ≥60% bone marrow monoclonal plasma cells, a kappa to lambda serum-free light chain ratio (involved chain/unaffected chain) >100 (involved chain must be present in excess of 100 mg/L), and ≥1 focal lesion on magnetic resonance imaging (MRI).

The reason for adding these three criteria into the definition of symptomatic MM lies in their prognostic value. In fact, certain parameters have been identified, listed above, that circumscribe a fraction of patients with SMM defined as at high risk of progression to MM, at an evolution of approximately 40% per year and therefore significantly higher than the 10% commonly reported in patients with SMM. For these high risk patients, it is believed, in the light of recent pharmacological developments, that it is necessary to establish treatments capable of preventing an almost certain evolution of myeloma from asymptomatic to symptomatic, while at the same time preventing the comorbidities that this evolution entails.

A Mayo Clinic study, conducted on a cohort of patients with SMM diagnosed between 1996 and 2010, described a sub-population equal to 3% of patients with bone marrow invasion by monoclonal plasma cells greater than or equal to 60%. At 2 years, 95% of these patients had progressed to asymptomatic smoldering myeloma, with a median time to progression of approximately 7 months [38]. A second study confirmed the data reported by Rajkumar et al. In a group of 96 patients with SMM, the median time to progression to symptomatic myeloma was 15 months for those with a plasma cell count ≥60% [39].

The normal ratio of kappa to lambda free light chains (K/L) measurable in serum is between 0.26 and 1.65; the presence of a monoclonal plasma cell population expressing one of the two K/L chains inevitably leads to an imbalance in the relationship between the two. Previously, it was reported by the Mayo Clinic team that an unbalanced K/L ratio, at least 8-fold higher, is associated with a 40% risk of progression from SMM to symptomatic MM in the first two years [40]. In a population of 586 patients with SMM, Larsen et al. defined a ratio of the involved to uninvolved serum free light chain ≥100, with a serum concentration of the involved light chain ≥100 mg/L as predictive of a progression to symptomatic MM or amyloidosis within 2 years in 82% of patients.

In addition, 27% of patients with a light chain ratio ≥100 developed acute renal failure due to myeloma progression-related end-organ damage [41]. These data were confirmed by Kastritis et al.; of 96 patients with SMM, 7% had a free light chain ratio ≥100, and almost all progressed within 18 months of the first observation [39].

In the staging and follow-up of MM, whether smoldering or active, MRI plays a fundamental role. In MRI, both diffuse anomalies and focal lesions can be detected. Hillengass et al. applied the whole-body MRI method to 196 patients with SMM, showing focal lesions in 28% of cases [42]; 15% of patients tested had >1 focal lesion. In this group of patients, the median time to progression was 13 months, and 70% of patients had undergone progression at 2 years. In confirmation of these data, Kastritis et al. published an analysis conducted on a group of 65 patients with SMM. In 14%, >1 lesion on MRI was highlighted. At 2 and 3 years, the rate of progression to symptomatic MM was 69% and 85%, respectively (median time to progression, 15 months). In patients with one or no focal lesions, the median time to progression exceeded 5 years [43].

For a correct classification of the disease, it is necessary to perform specific tests to define the quality and quantity of the monoclonal component, both on the serum and on the urine. It is therefore necessary to assess the following: electrophoretic protein pattern on serum proteins, dosage of serum immunoglobulins heavy chain components (IgA, IgG, IgM), dosage of free light chains in serum, immunofixation on serum and urine, and dosage of proteinuria and Bence-Jones proteinuria on 24-h urine [44].

The dosage of free light chains is recommended in any patient with a plasma cell disorder at diagnosis, especially in patients with: (a) non-secretory myeloma (absence of monoclonal component, 3% of all myeloma patients according to data published by the Mayo group Clinic) [45]; (b) small amounts of monoclonal component (oligosecreting myeloma); (c) myeloma secreting only light chains [46].

To complete the diagnostic workup, it is necessary to measure the following at diagnosis: complete blood count, liver and kidney function (serum creatinine and urea), serum calcium, lactate dehydrogenase (LDH) levels, beta-2-microglobulin (reflecting the ‘quantity’ or “disease burden”), and serum albumin.

The presence of plasma cells in the bone marrow is confirmed by bone marrow aspiration and bone biopsy. The percentage of plasma cells can be accurately measured using anti-CD138 antibodies, while clonality can be assessed by the identification of the cytoplasmic light chain. Furthermore, it is necessary to perform “fluorescent in situ hybridization” (FISH) for the evaluation of the chromosomal structure of the plasma cells under examination (preferably purified), using probes for the search for the following chromosomal alterations: del17p13, del13, del1p t(4;14), t(14;16), t(11;14), and amplification of chromosome 1q [47,48]. Conventional karyotype analysis provides additional information on plasma cell ploidy.

Bone involvement is a frequent feature of patients with myeloma, approximately 70–80% of whom have skeletal related events (SRE) at diagnosis [17,23,37]. The main examination for the detection of bone lesions was historically conventional radiography (X-ray) of the whole skeleton (skeletal system). The osteolytic lesions detectable by X-ray have the classic lytic appearance in the absence of a sclerotic border. The lesions are localized preferentially at the level of the vertebral column, ribs, skull, and pelvis [49]. However, in recent years, systematic diagnostic work-up began to include several radiological methods that have shown a greater sensitivity in identifying the presence of bone disease: whole-body low-dose CT (WBLD-CT), positron emission tomography (PET)-CT, and MRI. These methods were therefore included in the new myeloma diagnostic criteria published in 2014 as myeloma bone involvement detection techniques [35].

Unlike X-ray and CT, which can distinguish specific bone destruction from the invasion of plasma cells, MRI highlights bone marrow infiltration by myeloma cells. In order of frequency, five marrow invasion patterns have been described in MRI: (1) focal lesions >5 mm in diameter; (2) widespread invasion with total replacement of normal bone marrow tissue; (3) a mixed pattern with focal lesions and diffuse invasion; (4) normal marrow; (5) a salt and pepper pattern with countless minute focal lesions [49,50,51].

Preliminary data suggest that the typology of the MRI presentation pattern in patients with myeloma at diagnosis constitutes an independent prognostic factor (i.e., a diffuse infiltration pattern and a high number of focal lesions) [52,53].

MRI therefore plays a fundamental role in the staging of the patient with SMM for a correct clinical definition, in staging the newly diagnosed patient with symptomatic myeloma, especially if X-ray or WBLD-CT for bone involvement is negative, and in the staging of patients with a solitary bone plasmacytoma. Furthermore, MRI is a fundamental examination both for discriminating between osteoporotic or myeloma-related vertebral sagging and for accurately describing any compression of the marrow or nerve roots, an essential aspect for a possible surgical approach. The role of MRI in patient follow-up and in defining bone response to therapy is still the object of study and debate.

PET-CT is an instrumental examination that combines the identification of bone lesions by CT with the functional evaluation of the metabolic activity of tumor cells. First, PET-CT has been shown to be useful in staging myeloma, as it has a greater sensitivity than conventional radiography in identifying osteolytic lesions. Second, PET-CT has proven to be effective in identifying extra-medullary disease (EMD), both as a predictor of the evolution of asymptomatic forms into symptomatic myeloma and as a prognostic factor during therapy [54].

In a study of 188 SMM patients, 39% had a positive PET-CT, with a 2-year progression rate to symptomatic myeloma of 75%, compared with 30% of patients with a negative PET-CT. In another study, conducted on patients with previously defined asymptomatic myeloma, 16% of patients with a negative systematic skeletal radiograph for osteolytic lesions had a positive PET-CT. The median time to progression to symptomatic myeloma of these patients was 1.1 years, significantly less than the 4.5 years for patients with a negative PET-CT [55,56]. For this reason, in the recent IMWG myeloma diagnostic criteria, the presence of positive lesions in PET-CT is a sufficient criterion to implement chemotherapy. Analysis of the metabolic activity of the disease using PET-CT has been shown to delineate a statistically significant prognostic factor both at diagnosis and in the context of monitoring the response obtained with therapy [57,58]. Extensive uptake, high uptake intensity (in terms of the Standardized Uptake Value, SUV), and the presence of EMD at diagnosis are unfavorable prognostic elements. As regards the evaluation of the response to therapy, signal suppression is correlated with the biochemical response achieved after chemotherapy. Persistence of PET-CT positivity is significantly associated with a shorter survival. For this reason, PET-CT assessment of minimal residual disease (MRD) has been combined with bone marrow MRD assessment in the IMWG criteria published in 2016 [59].

This method presents promising results, but still needs to be suitably standardized. An Italian group has recently published new interpretation criteria for PET-CT images that will be validated in randomized trials [60].

According to the most recent guidelines, all patients with suspected myeloma should be examined for bone involvement by WBLD-CT, a method that has replaced systematic skeletal radiography. If WBLD-CT is unavailable, conventional radiography remains the first level investigation. MRI, preferably whole-body MRI, is indicated in patients with SMM and in those with MM with negative level I radiographic examinations, as well as in the case of spinal cord compression or EMD of bone origin. PET-CT is useful in evaluating EMD and in defining the response to therapy but depends on the availability of this resource.

## 3. Risk Stratification

The individual prognosis of MM is mainly linked to two categories of risk factors: those related to the patient and those connected with the intrinsic biological characteristics of the disease. Patient-related factors are age, comorbidities, and clinical condition (“fitness” or frailty). Prognostic factors related to MM biology are albumin and beta-2 microglobulin, which represent the burden and cytogenetic abnormalities present in myeloma plasma cells.

Historically, patients with MM were categorized according to Durie and Salmon staging, which subdivides patients into three stages with a progressively worse prognosis based on clinical data, such as the extent of the monoclonal component and the presence or absence of signs of organ damage [61]. Over 70% of patients were in stage III, and the predictive ability in individual patients was limited. In recent years, this classification was progressively replaced by the International Staging System (ISS) and some ISS revisions (R-ISS, R2-ISS) [35,62,63,64]. The ISS is smart and simple and takes only two serum parameters into consideration: beta-2 microglobulin, closely linked to renal function and tumor mass, and albumin, thus defining 3 risk classes (ISS 1, 2, and 3). Moving on to R-ISS, cytogenetic risk was introduced by defining high risk del17p, t(4;14) and t(14;16), and LDH. Ultimately, the R2-ISS was developed, defining 1q amplification as high risk cytogenetics. The R2-ISS, which was introduced in 2019, is an updated version of the R-ISS, and incorporates additional cytogenetic abnormalities as high-risk factors. Specifically, the R2-ISS includes the presence of 1q21 amplification as a high-risk cytogenetic abnormality in addition to the abnormalities included in the R-ISS. The addition of 1q21 amplification as a high-risk factor in the R2-ISS was based on multiple studies that have shown its association with poor outcomes in multiple myeloma patients, including shorter progression-free survival and overall survival [63]. The R2-ISS is currently the most comprehensive staging system for multiple myeloma and is widely used in clinical practice to guide treatment decisions and predict patient outcomes. 

Alongside ISS, certain cytogenetic abnormalities are among the strongest prognostic factors described to date. The presence of the 17p13 deletion (on which the tumor suppressor TP53 is located), del1p deletion, t(4;14) translocation, and 1q21 amplification are chromosomal abnormalities that confer a poor prognosis. Translocation t(14;16) and the deletion of chromosome 13 appear to be correlated with a poor prognosis, although clear data are lacking. Translocation t(11;14), instead, represents an anomaly with a favorable prognosis [65]. In addition to cytogenetic data, data are emerging in recent years about the prognostic power of specific “gene expressions”. Studies in progress are evaluating “patterns of gene expression” whose role in clinical practice, however, remains to be defined [66,67,68]. In this setting, a new clinical entity, namely double hit myeloma, has been recently described. It consists in a bi-allelic TP53 inactivation and identifies very high risk patients associated with poor prognosis and PFS despite novel treatments [67].

Age has long been the fundamental criterion for patient eligibility for high-dose chemotherapy and an autologous transplant procedure, as an alternative to less intensive or even palliative therapy. However, it should be noted that aging is not a homogeneous biological phenomenon. Recently, much evidence has emerged regarding the need to integrate age with elements of assessment of the patient’s fitness, thus moving away from the concept of chronological age and toward that of biological age. An IMWG study conducted on 869 patients, enrolled at the diagnosis of myeloma in three experimental protocols with new drugs, led to the creation of a geriatric score combining parameters such as age with parameters deriving from the application of tools for assessing comorbidity (Charlson Comorbidity Index) and patient fitness (ADL—Activities of Daily Living, and IADL—Instrumental Activities of Daily Living). This score, known as the IMWG frailty score, proved to be able to stratify the study patients into three groups (fit, unfit, and frail), each with different risks of progression, death, and incidence of treatment-related toxicity [69,70].

## 4. Modern Therapeutic Approach to Multiple Myeloma

Nowadays, only patients with symptomatic MM require chemotherapy treatment. Patients with SMM, even those at high risk of progressing to symptomatic MM, should not be treated outside clinical trials.

Before defining the therapeutic approach, it is necessary to describe the clinical history of MM itself. Once the diagnosis of symptomatic MM has been made, and therefore the need to establish anti-myeloma treatment has been ascertained, the patient is treated with what is defined as first line of therapy (LOT). Based on the effectiveness of the chosen treatment, a period of remission may be achieved and maintained. Almost inevitably, however, small amounts of the plasma cell clone survives and proliferates again, thus configuring a picture of recurrence. The latter is defined as “biochemical” in the presence of only plasma cell proliferation at the medullary level and the consequent increase in the blood and urine of its specific marker, i.e., the monoclonal component produced by the plasma cells, or as “clinical” when this proliferation is accompanied by damage to the body, ranging from anemia through bone lesions to renal failure and up to hypercalcemia. The time interval between the initiation of therapy and recurrence is termed progression-free survival (PFS).

When MM relapses, a new LOT must be established. The IMWG (International Myeloma Working Group) guidelines recommend treating the patient in cases of clinical recurrence or “aggressive” biochemical recurrence, the latter being characterized by a rapid increase in the monoclonal component, conceived as an expression of rapid cancerous proliferation.

The adoption of a subsequent LOT that will produce effective results is therefore followed by a new period of remission, the length of which is highly variable and dependent on various factors. Consequently, the clinical history of myeloma is characterized by an alternation of latency and recurrence phases, which connote a chronic course of the disease.

Until the early 1990s, the therapeutic background of MM envisaged the use of classic chemotherapeutic agents—such as melphalan and cyclophosphamide, the vinca alkaloids, such as vincristine, and anthracyclines, such as idarubicin, in combination with steroids at high doses—and the myeloma patient’s prognosis was poor, with median overall survival (OS) being 2–3 years. The first revolution in the treatment of MM came with the introduction of autologous transplantation, a procedure containing high dose alkylating agents, melphalan, followed by the support of previously collected autologous stem cells to allow bone marrow reconstitution. This procedure made it possible to significantly increase the percentage of patients who achieved complete remission (CR), or otherwise sporadic, and concomitantly produced better long-term disease control, with an increase in OS. However, this improvement only benefited patients eligible for transplantation, i.e., the “younger” and the “fitter” ones. In the early 2000s, the era of new drugs began, of which thalidomide is the progenitor. This era was characterized by a deeper knowledge of the biology of MM and of the microenvironment in which myeloma cells proliferate (the hematopoietic marrow), as well as by the consequent development of targeted drugs with specific different mechanisms of action, but all aimed at affecting cancer cells. The new drugs can be used for both the treatment of younger patients and candidates for transplantation and for that of older patients, previously ineligible for this procedure. Since the early 2000s, the US (Food and Drug Administration—FDA) and European (European Medicine Agency—EMA) regulators have approved more than ten drugs of different classes, including immunomodulatory drugs, proteasome inhibitors, monoclonal antibodies, bispecific t-cell engagers (BiTE), and chimeric antigen receptor T cells (CAR-T) for the treatment of multiple myeloma, some of which are licensed in single administration, others in combinations. 

The therapeutic approach to patients with multiple myeloma essentially depends on two elements: the stage of the disease (diagnosis or recurrence) and the patient’s eligibility for autologous transplantation, a condition defined based on chronological age and clinical conditions (comorbidities, organ damage, absence of fragility). By convention, the patient who is a candidate for high-dose chemotherapy and autologous stem cell transplantation is defined as “young”, while the patient who is not a candidate for this procedure is defined as “elderly”. Traditionally, the age limit that served as a watershed between the young and the elderly patient was 65 years. This age limit, however, has been raised over time: currently, in Europe patients up to 70–75 years of age, in adequate clinical conditions, are considered potential candidates for high-dose chemotherapy and a transplant procedure, while in the United States United States this limit was recently further extended up to 79 years [71].

### 4.1. Newly Diagnosed MM: Autologous Transplant Candidate

Despite the introduction of new drugs, high-dose chemotherapy (melphalan 200 mg/m^2^), followed by autologous hematopoietic stem cell transplantation, remains the standard in the patient who is judged suitable for this procedure. However, this approach is currently challenged by various new therapeutic strategies involving CAR-T or BiTE.

Several studies have compared therapeutic strategies for first LOT in multiple myeloma based on the use of new drugs with or without autologous transplantation. In all these studies, it has been demonstrated that incorporating autologous transplantation into first LOT allows a significant prolongation of PFS. Moreover, some of these studies also demonstrated an overall survival (OS) advantage in favor of transplantation [31,72,73,74]].

The first LOT in the patient eligible for autologous transplant consists of five different phases: induction therapy, stem cell mobilization, autologous transplant (single or double), consolidation therapy, and, finally, maintenance therapy.

Induction therapy aims to reduce the tumor mass of proliferating plasma cells at the time of diagnosis, reducing or eliminating the organ damage related to the onset of MM and allowing the medical staff to proceed with stem cell collection without interfering with the mobilization of the stem cells, and allowing the subsequent transplantation with the lowest possible degree of residual disease. The current standard induction regimen in Europe and the United States is based on the combination of bortezomib (V), a proteasome inhibitor (PI), an immunomodulator (IMiD) such as thalidomide (T, in Europe) or lenalidomide (R), and dexamethasone (D) (VTD, VRD). Recently, the Phase III CASSIOPEA and Phase II GRIFFIN trials demonstrated that the addition of a fourth drug, the anti-CD38 monoclonal antibody daratumumab (D), to the VTD (D-VTD) and VRD (D-VRD) triplets significantly increases the response rate, including the rate of patients achieving MRD negativity, and PFS (in the case of the CASSIOPEIA study) when compared to the triple regimen. D-VTD and D-VRD have been approved by the FDA and EMA, respectively, and are, therefore, the new standard of care for patients with newly diagnosed MM who are eligible for autologous transplantation [75,76,77]. These regimens allow, at the end of the first four induction cycles, at least partial responses to be obtained in over 90% of patients [78,79].

The second phase of the first LOT consists of the mobilization and collection of stem cells. This procedure can be preceded by the administration of chemotherapy capable of stimulating the “leakage of stem cells”, such as cyclophosphamide (dosage 2–4 g/m^2^). Moreover, the administration of the granulocyte growth factor known as G-CSF (granulocyte-colony stimulating factor) stimulates the proliferation and leakage of CD34+ stem cells into the peripheral blood. The CD34+ cell count indicates the optimal moment for apheresis, i.e., the opportune moment for their collection, which today is performed from peripheral blood in 1–2 consecutive apheresis sessions. Prior to the introduction of plerixafor, a type 4 chemokine receptor antagonist, the rate of patients defined as poor mobilizers, i.e., in whom it was not possible to harvest an adequate number of CD34+ cells to allow subsequent autologous transplantation (≥2 × 10^6^/Kg), was equal to 5–15% of patients who underwent hematopoietic stem cell stimulation and apheresis. The introduction of plerixafor, used by default as a mobilization regimen together with G-CSF, or as needed, “on demand”, in patients with poor peripheral mobilization of stem cells after the administration of G-CSF or chemotherapy and G-CSF, has made it possible to significantly reduce the rate of “poor mobilizer” patients [80]. Currently, patients deemed candidates for autologous transplantation receive one or two cycles of high-dose chemotherapy followed, at each chemotherapy cycle, by reinfusion of previously harvested stem cells. It is therefore important that the apheresis collection at the end of the induction therapy is sufficient to allow at least two stem cell transplants.

The third phase of first LOT is autologous stem cell transplantation. The patient is administered a dose of melphalan equal to 200 mg/m^2^ (in case of renal failure or significant comorbidities, the dose of melphalan can be reduced to 100–140 mg/m^2^) and then infusion of previously harvested stem cells after 24–48 h. The administration of high doses of melphalan is considered myeloablative, i.e., capable of killing bone marrow stem cells in a potentially irreversible manner. The infusion of autologous stem cells allows a rapid bone marrow reconstitution. Otherwise, the phase of aplasia following melphalan would be burdened by a high mortality caused by infections linked to the low white blood cell count and by hemorrhages resulting from thrombocytopenia. Stem cell transplantation can be repeated a second time, 3–6 months after the first, in order to obtain the maximum possible cytoreduction. The benefit of double transplant was initially seen in patients who did not achieve at least one VGPR after the first transplant [81]. The analysis was conducted on patients enrolled in the HOVON65/GMMG-HD4 study in the context of treatment with bortezomib, both in induction and maintenance. Although the study was not designed for this comparison, it showed a survival advantage for patients receiving two transplants when compared to patients receiving one transplant [82]. A study recently conducted by the European Myeloma Network (EMN) demonstrated a PFS advantage for double transplant when compared to single transplant [31], particularly in patients considered to be at very high risk through the presence of FISH abnormalities such as del17p, t(4;14) or double hit myeloma [31]. Similarly, the benefit of a double transplant when compared to a single transplant was observed, in terms of PFS, in patients at high cytogenetic risk in the phase III StaMINA study [83]. By contrast, in patients at standard risk, there was no difference in terms of PFS and OS between a single and a double transplant in a long-term follow-up analysis of three phase III trials [84]. As such, double autologous transplantation can currently be considered in high-risk patients and in the presence of high-risk cytogenetic alterations. Additionally, they need to show good tolerance and evidence of a clinical benefit from the first transplant.

Following the transplant, the patient may receive a limited number of cycles of therapy of equal or similar intensity to that of induction therapy (generally two) in order to further reinforce the response obtained with the transplant. This phase of the first LOT in patients eligible for autologous transplantation is called consolidation. Several studies that have incorporated this approach into the overall treatment strategy of the newly diagnosed MM patient have shown a progressive strengthening of responses from the induction phase, through transplantation to consolidation. The regimens that have shown such efficacy are VTD, VRD, DVTD, DVRD [72,78,79,85,86,87], and KRd [88]. However, these studies do not allow the real benefit of consolidation therapy to be established as they do not provide for a randomization of patients enrolled in a consolidation arm when compared with an arm without consolidation. Therefore, there are conflicting data regarding the benefit of consolidation. The EMN02/H095 study demonstrated, through a formal randomization, a benefit in terms of PFS for patients who, after the first phase of treatment, received a limited number of cycles of consolidation, according to the VRD scheme, when compared to patients who continued directly with the maintenance therapy. The StaMINA study [83], on the other hand, in which patients with newly diagnosed MM, after an initial induction phase and autologous transplant, were randomized to a second transplant followed by maintenance lenalidomide, consolidation with VRD and lenalidomide maintenance, or directly to maintenance lenalidomide, did not demonstrate a greater benefit when compared to a short course of consolidation before maintenance therapy. The use of consolidation therapy in MM, therefore, remains the subject of debate, although many experimental clinical studies have incorporated it. The newly approved regimen DVTd, as well as DVRd upon its approval by EMA, includes two further cycles of consolidation with the same drugs used during the induction phase.

The last phase of the first line treatment is maintenance therapy, which must be effective in maintaining and/or improving the response obtained and in prolonging the remission period and overall survival. Maintenance should be easily administered to the patient, well tolerable, and should not interfere with the patient’s quality of life. The first drug to demonstrate an advantage in terms of PFS as post-transplant maintenance was thalidomide [89]. However, this benefit did not extend to overall survival (OS). Moreover, the poor tolerability of thalidomide, burdened by high rates of peripheral neuropathy, negatively affects the long-term administration of this drug. The thalidomide analog lenalidomide has been extensively tested as autologous post-transplant maintenance. Several phase 3 studies have demonstrated a PFS benefit for patients who received lenalidomide as a post-transplant maintenance agent [90,91,92,93]. A meta-analysis that examined 1200 patients included in the aforementioned trials showed that the survival advantage for patients who received lenalidomide also extended to OS, conferring an advantage of about 2.5 years when compared to patients who received lenalidomide but not maintenance therapy, or else received placebo [94]. Based on these data, lenalidomide was approved by both the FDA and the EMA as a maintenance drug in patients who had previously undergone autologous stem cell transplantation. The duration of maintenance with lenalidomide is heavily discussed; to date, it is indicated until progression or intolerance. No data are available on the possibility of interrupting treatment when a certain period of treatment or a specific response to therapy is reached (e.g., in CR patients). Lenalidomide also forms the basis on which two- or three-drug combinations, including ixazomib, bortezomib, carfilzomib, daratumumab, and isatuximab, have been tested or are being tested as post-transplant maintenance therapy. For example, in the randomized FORTE study, the addition of carfilzomib to lenalidomide as post-transplant maintenance therapy demonstrated a prolongation of PFS when compared to lenalidomide as monotherapy [95].

Bortezomib has been tested as a maintenance agent in both young post-transplant patients, and in elderly patients at the end of induction therapy. The phase III HOVON65/GMMG4 study compared bortezomib and thalidomide as maintenance therapies in patients undergoing autologous transplant, demonstrating a survival advantage in favor of bortezomib, even in patients considered to be at high risk based on cytogenetic characteristics (FISH), particularly those who presented del17p or t(4;14). However, the main limitation of the study was the different induction regimens in the two arms, which did not allow the evaluation of the benefit of continuous bortezomib therapy compared to thalidomide. Therefore, bortezomib is not approved as post-transplant maintenance, but could be considered in individual high-risk cases [82]. However, several guidelines recommend an approach to HR patients that also includes bortezomib. Its parenteral administration, as well as the risk of peripheral neuropathy, have limited its development as a therapy for continuous administration. More recently, the phase III Tourmaline-MM3 study enrolled 702 patients with newly diagnosed MM undergoing a single autologous transplant, with the aim of evaluating the efficacy and safety of ixazomib, a proteasome inhibitor analog of bortezomib but with oral bioavailability; ixazomib was administered for 26 cycles compared to placebo as maintenance therapy post-autologous transplantation [96]. This study demonstrated a statistically significant reduction, in favor of ixazomib, in the risk of progression or death (HR: 0.72; *p* = 0.002) when compared to placebo. This benefit was also extended to the subgroup of patients at high cytogenetic risk.

Despite the benefits of maintenance therapy, recurrence is almost inevitable. To improve the efficacy of maintenance therapy, several studies are underway to test two-drug maintenance regimens, combining lenalidomide with proteasome inhibitors (carfilzomib and ixazomib) or monoclonal antibodies (daratumumab and isatuximab) or proteasome inhibitors with monoclonal antibodies (ixazomib and daratumumab). Daratumumab, a monoclonal antibody directed against plasma cell-expressed CD38, has also been used as a drug for post-transplant maintenance therapy. In the phase III CASSIOPEIA study, patients with first-line myeloma treated with bortezomib, thalidomide, and dexamethasone (VTd), with or without daratumumab as induction and consolidation therapy as part of a transplant strategy, were randomized to maintenance therapy with daratumumab for two years or observation alone. The authors reported a statistically significant PFS benefit (47% reduction in the risk of progression or death) for patients who received maintenance daratumumab when compared to patients in the control arm [97].

Allogeneic transplantation is considered a potentially curative option for young patients with a compatible donor. However, chronic graft versus host disease (GVHD) remains the leading morbidity, with a major impact on patient survival and quality of life. In a randomized study, conducted in the era before the introduction of new drugs, allogeneic transplantation was superior to autologous transplantation [98,99]. Following the introduction of new drugs and the results obtained with them, allogeneic transplantation is considered a less attractive option and its use is not recommended outside clinical protocols. Its use remains confined to randomized trials, in association with new drugs, in very high-risk patients (e.g., deletion of chromosome 17), particularly in patients with early recurrence after autologous transplantation [100].

### 4.2. Newly Diagnosed MM Not Eligible for Autologous Transplant

The introduction of geriatric scores capable of identifying precise subpopulations within the group of elderly patients has allowed the development of clinical studies aimed directly at specific subgroups of patients to test, in a precise and targeted manner, the efficacy and safety of therapeutic regimens. In one of these studies, aimed at testing chemotherapy regimens in specific elderly populations, Larocca A. et al. demonstrated that, in patients defined as “unfit” through the frailty score of the International myeloma working group and treated with lenalidomide and dexamethasone (Rd), the reduction of the lenalidomide dosage and the suspension of dexamethasone after a full-dose induction phase did not determine a reduction in the efficacy of the combination when compared to its use at a standard dose [101].

Since the 1960s, the combination of orally administered melphalan and prednisone (MP) has been the standard treatment of the newly diagnosed MM patient. With the advent of autologous transplantation, this combination was reserved for patients ineligible for transplantation. However, the therapeutic approach to myeloma changed radically when, at the beginning of the year 2000, the first “novel agent”, namely thalidomide, was introduced. Since then, a series of anti-myeloma drugs have been developed, tested, and then introduced into clinical practice and incorporated into the therapeutic strategies for elderly patients. Specifically, the main players in this revolution have been thalidomide, lenalidomide, and bortezomib before, and then daratumumab.

The first revolution in the treatment of the elderly patient was the addition of thalidomide to the standard MPT (MPT). Palumbo A et al. first reported that the MPT combination, when compared with the standard MP, led to a significantly higher rate of CR (27.9% vs. 7.2%) and an increase in PFS (2.8 vs. 14.5 months, *p* = 0.004). However, an OS advantage in favor of thalidomide-treated patients was not demonstrated (40.5 vs. 47.6, *p* = 0.79) [102,103].

Several randomized studies have confirmed the benefit of adding thalidomide to the MP combination alone, both in terms of response and PFS, but not all studies have also shown a survival advantage [102,103,104,105,106,107]. A meta-analysis based on previously conducted studies demonstrated that the MPT triplet is associated with a higher response rate, longer PFS, and a trend toward better survival when compared to the MP doublet [108].

Considering the new combinations available, such as lenalidomide-dexamethasone (Rd), associated MP+bortezomib (VMP), or daratumumab and VRd, the MPT scheme is no longer a currently valid therapeutic option as first-line therapy for patients who are not candidates for autologous transplantation.

The new guidelines of the European Society of Medical Oncology (ESMO), updated in 2021, recommend which combinations of 1st line therapy are suitable for the patient not eligible for DRd, DVMP, or VRd autologous transplant. Instead, the second choice options are Rd and VMP [75].

The addition of bortezomib to the combination of MP (VMP) was investigated as first-line therapy in patients over 65 years, or younger but not eligible for autologous transplant, and compared with standard MP. In this randomized study, 682 patients affected by MM at diagnosis were enrolled and randomized to the two treatment arms (VMP vs. MP). Among patients treated according to the VMP regimen, there was a higher rate of CR (30% vs. 4%, *p* < 0.001) and an increase in both time to progression (TTP; median, 24 vs. 16.6 months, *p* < 0.001) and OS (median, 56.4 vs. 43.1 months, *p* < 0.001). Based on these data, the VMP scheme was approved as 1st line therapy for patients ineligible for autologous transplantation [109,110].

The ALCYONE study randomized 706 newly diagnosed MM patients, ineligible for autologous transplantation, to receive standard therapy, i.e., nine cycles of PVD (pomalidomide, bortezomib, dexamethasone) or nine cycles of PVD plus the anti-CD38 monoclonal antibody daratumumab followed by maintenance therapy up to progression with daratumumab alone. In this study, the use of a four-drug regimen (DVMP- daratumumab, bortezomib, melphalan, prednisone) as induction therapy and continuous administration of daratumumab as maintenance therapy resulted in a statistically significant reduction in the risk of progression or death (HR: 0.42; *p* < 0.0001) and that of death (HR: 0.6; *p* = 0.003) when compared to the VMP standard regimen [111,112]. DVMP also demonstrated a significant increase in the percentage of patients achieving negative minimal residual disease (MRD) when compared to VMP (27% vs. 7%, sensitivity 10^−5^) [111].

Lenalidomide, a second-generation immune-modulator derived from thalidomide was tested in patients with newly diagnosed MM after demonstrating its efficacy in patients with relapsed and/or refractory MM [113,114]. The US randomized phase III ECOG E4A03 trial tested lenalidomide in patients with newly diagnosed MM, including transplant ineligible patients, and compared high-dose (RD) versus low-dose (Rd) dexamethasone use. The use of low-dose dexamethasone, in combination with lenalidomide, demonstrated an OS advantage, particularly evident in patients >65 years of age [115].

A formal comparison between standard of care MPT and Rd was conducted in the FIRST study, a randomized phase III trial, which enrolled 1623 patients with previously untreated MM and ineligible for autologous transplantation. Patients were randomized to three treatment groups: MPT, Rd for 18 months, and continuous Rd until progression or intolerance. The primary endpoint of the study was PFS, while secondary endpoints were OS and adverse events. After a median follow-up of 37 months, patients treated with continuous Rd had a PFS advantage with a 28% reduction in progression or death compared with patients randomized to MPT (HR, 0.72; 95% CI, 0.61–0.85, *p* < 0.001) and 20% versus patients treated with Rd for 18 cycles (HR, 0.70; 95% CI, 0.89–1.20; *p* = 0.70). An OS advantage was also demonstrated for patients in the continuous Rd arm when compared to patients in the MPT arm (HR: 0.78; CI, 0.64–0.96, *p* = 0.02) [116].

The US randomized phase III SWOG S0777 study compared adding bortezomib to the Rd combination (VRd) versus using Rd alone in 525 patients with newly diagnosed MM, without the intent to proceed with autologous transplantation [117]. Of these, just over half were aged 65 or over. This study demonstrated that the use of the VRd triplet induces a higher rate of ORR (82% vs. 72%), with a statistically significant advantage in terms of both median PFS (41 vs. 29 months) and median OS (not reached vs. 69 months) when compared to the Rd doublet [118]. Based on these data, VRd represents a standard of care for the elderly patient in the United States and in Europe.

In the randomized MAIA study, we evaluated the efficacy and safety of adding daratumumab to the Rd doublet versus standard Rd administration until progression or intolerance in newly diagnosed elderly patients with MM. As reported for the D-VMP combination in the ALCYONE study, the addition of daratumumab resulted in an increased rate of CR (47.6% vs. 24.9%) as well as patients with negative minimal residual disease in this clinical trial (29% vs. 9%, 10^−5^) [119,120]. The better depth of response obtained with DRd compared to Rd also translates into a significant reduction in the risk of progression (HR: 0.53; *p* = 0.0013) and death (HR 0.68; *p* < 0.001), to the advantage of DRd [120].

Several studies have investigated the effect of maintenance therapy after one induction phase with new drugs in newly diagnosed MM patients at the end of induction therapy (MPR-R vs. MPR vs. MP [121], VMP-VP vs. VTP-VT [122]). However, the nature of the studies mentioned above did not allow the approval of these drugs as maintenance in patients not eligible for transplantation. In the phase III TOURMALINE-MM4 study, conducted on 706 patients with a median age of 72 years and not eligible for autologous transplantation, patients were randomized to receive maintenance therapy for about 2 years with ixazomib or placebo. In patients randomized to the experimental arm, ixazomib significantly reduced the risk of progression or death by 31.4% (HR: 0.659; *p* < 0.001) when compared with placebo; median PFS from the start of maintenance therapy was 17.4 months in the ixazomib arm and 9.4 months in the placebo arm. Another important finding is that the efficacy of ixazomib in prolonging median PFS was observed in all subgroups of patients analyzed, regardless of age, frailty score, and stage of disease (ISS). A longer follow-up will allow an evaluation of the impact of ixazomib on OS [123].

The need to identify frail patients in recent years stems from both poorer outcomes and an increased risk of therapy discontinuation displayed by frail patients. This led to the development of novel tools, among which geriatric assessment, which evaluates daily activities and comorbidities through the *Activities of Daily Living* Scale (ADL), Instrumental ADL Scale (IADL), and Charleston Comorbidity Index (CCI). The introduction of these tools into the IMWG frailty score has been recently proposed [70]. Identification of the patients who may benefit from therapy should be pursued in order to tailor treatments to the patients’ features and comorbidities, improve PFS, reduce treatment toxicity, and improve quality of life. Indeed, a retrospective frailty assessment in both the ALCYONE [124] and MAIA [125] trials showed a higher PFS in non-frail patients when compared to frail patients. Moreover, treatment toxicity, which stems from an increased risk for early severe infections, has been observed in frail patients, negatively affecting PFS [126]. Frail patients tend to show a relatively lower health related quality when compared to fit patients [127]. In addition, data suggest that optimization of treatment in frail patients by a steroid sparing regimen achieved a better overall response rate, higher MRD negativity, and lower rates of infection [128]. Nonetheless, their use has been disputed by experts, given the limitations to these scores, including time-consumption, role of age, and performance status. Therefore, current assessment scores are deemed unsuitable for clinical practice and not specific for MM. New tools (i.e., biologic markers) and improvement of discriminative power of current scores should be able to stratify patients not only by OS but also by impact on QoL and on treatment choice [129].

### 4.3. Therapeutic Approach to the Patient with Relapsed/Refractory Myeloma

Until 2015, the only new drugs approved by EMA, and therefore available for patients with relapsed and/or refractory MM (RRMM) after at least one line of therapy, were lenalidomide, in combination with dexamethasone (Rd), and bortezomib, both as single agent and in combination with dexamethasone and/or pegylated doxorubicin. In randomized phase III studies, both lenalidomide and bortezomib in combination with dexamethasone, were shown to be superior in terms of PFS when compared to the administration of dexamethasone alone, thus representing the standard of therapy of RRMM patients for several years after a first line of therapy [113,114,130,131,132,133,134].

Recently, however, a deeper knowledge into myeloma biology and the development of new molecules with demonstrated anti-myeloma efficacy (both in vitro and in vivo) have led to clinical trials testing these drugs as single agents or combined with immunomodulators and proteasome inhibitors. This allowed treatment to bypass the molecular resistance developed after the first line of treatment thanks to their different mechanism of action and targets. These drugs may offer patients with RRMM due to intrinsic and extrinsic mechanisms [135,136,137,138] effective therapeutic alternatives in restoring the disease to a state of clinical and biochemical latency.

The ESMO guidelines propose a choice of therapy for recurrence based primarily on the type of therapeutic combination introduced at diagnosis and on the patient sensitivity or refractoriness to lenalidomide, bortezomib, and daratumumab, i.e., the current cornerstone drugs of first-line therapy.

If the patient was treated with bortezomib at diagnosis, the first choice of therapy at relapse will be combinations containing lenalidomide and dexamethasone (Rd) plus a third drug such as daratumumab (DRd, if the patient was not treated with daratumumab for maintenance or not refractory), carfilzomib (KRd), ixazomib (IRd), or elotuzumab (ERd). If the patient was, instead, treated with Rd until progression, the therapeutic alternatives at the first relapse are based on the adoption of regimens containing a proteasome inhibitor, such as bortezomib or carfilzomib, associated with a monoclonal antibody, such as daratumumab (DVd, DKd) or isatuximab (IsaKd), or an immunomodulator, such as pomalidomide (PVd). Based on the previous lines, in the second relapse, combinations containing pomalidomide or carfilzomib are recommended, such as those already approved for the first relapse, in addition to isatuximab, pomalidomide and dexamethasone (IsaPd), and elotuzumab, pomalidomide, and dexamethasone (EPd). In case of multiple refractoriness to immunomodulatory drugs, proteasome inhibitors and anti-CD38 monoclonal antibodies, the antibody conjugate belantamab-mafodotin and the EXPO1 inhibitor selinexor, are two EMA approved options.

For years, the Rd combination has been the standard at relapse, particularly for patients treated with bortezomib at diagnosis. Investigators in the Phase III ASPIRE study compared the triplet of Rd + carfilzomib (KRd) with Rd in patients with RRMM after 1∓3 prior lines. The combination of a second generation PI, carfilzomib, with lenalidomide resulted in a statistically significant increase in ORR (87.1% vs. 66.7%; *p* < 0.001) and CR (31.8% vs. 9.3%), which is reflected in the prolongation of both median PFS (26.3 vs. 17.6 months, HR 0.69, *p* = 0.001) and median OS (48.3 vs. 40.3 months, HR 0.79, *p* = 0.01) [139].

The introduction of a new orally administrable proteasome inhibitor, ixazomib, allowed the development of the first oral combination of a PI with an IMID. This combination (IRd) was compared with the standard Rd in a phase III study, TOURMALINE-MM1, which enrolled patients with RRMM and no more than three prior lines of therapy. A statistically significant increase in median PFS was observed for patients in the IRd arm when compared to control (20.6 vs. 14.7, HR 0.74, *p* = 0.01) [140]. The final study analysis showed no differences in terms of OS between the two arms (median OS, 53.6 months vs. 51.6 months; HR: 0.939). However, a reduction in the risk of death was observed in patients with high cytogenetic risk (HR: 0.86) and in patients refractory to the previous line of therapy (HR: 0.74) [141].

Elotuzumab was the first monoclonal antibody introduced and approved for the treatment of MM. It is an IgG monoclonal antibody directed against signaling lymphocytic activation molecule F7 (SLAMF7), targeting it with an immunostimulatory activity. The additive effect and synergy between elotuzumab and lenalidomide was tested in the phase III ELOQUENT-2 study, in which patients with RRMM (1–3 prior lines) were randomized to EloRd vs. Rd. Patients included in the study had to be refractory to last line therapy and could have previously received lenalidomide, but were not required to be refractory to it. The study demonstrated a statistically significant advantage in favor of the EloRd arm over the Rd arm, both in terms of median PFS (19.4 vs. 14.9 months, HR 0.70; *p* < 0.001) and median OS (48.3 vs. 39.6 months, HR 0.82; *p* = 0.04) [142,143].

Daratumumab is a fully humanized (IgG4) antibody directed against the surface molecule CD38, widely expressed by plasma cells. Daratumumab was initially tested and then approved as a single agent in highly pre-treated RRMM patients. In the pooled analysis, which combined data from two different studies of daratumumab as monotherapy (patient cohort with a median of five prior lines of therapy), it induced at least a partial response in 31.1% of patients, with a median duration of response (DOR) of 7.6 months, and median PFS and OS of 5 and 20.5 months, respectively [144,145,146].

Once again, Rd represented the standard control arm against which the triplet, consisting of daratumumab + Rd (DaraRd), was tested in a phase III study (POLLUX). This study enrolled 569 patients with RRMM after at least one prior line of therapy, including lenalidomide in the absence of ongoing progression. Adding daratumumab to the Rd doublet demonstrated significant increases in ORR (93% vs. 76%, *p* < 0.001), CR rate (57% vs. 23%, p < 0.001), and MRD patients negativity (30% vs. 5%, sensitivity of 10^−5^, *p* < 0.001), then translating into an increase in median PFS in favor of DRd (44 vs. 17.5 months; HR 0.44, *p* < 0.0001) [147,148].

For the first time, the ENDEAVOR study directly compared two proteasome inhibitors (PIs), carfilzomib (Kd) and bortezomib (Vd), both associated with dexamethasone for the treatment of RRMM patients with 1–3 prior lines. Enrolled patients could have been previously treated with bortezomib but were not required to be refractory to it. Patients enrolled in the Kd arm benefited from an advantage in both PFS (median, 18.7 vs. 9.4 months, HR 0.53; *p* < 0.0001) and OS (median, 47.6 vs. 40.0 months, HR 0.79, *p* = 0.01) [149]. However, a further step forward in terms of efficacy was obtained by combining Kd with an anti-CD38 monoclonal antibody.

The IKEMA phase 3 study compared Isa-Kd to Kd as salvage treatment for patients with 1–3 prior lines of therapy, demonstrating a 47% reduction in the risk of death or progression in favor of the Isa-Kd triplet over the Kd doublet (median PFS, not reached vs. 19 months; HR 0.53; *p* = 0.0007). Even for lenalidomide-refractory patients, there was a 40% reduction in the risk of death or progression when compared with Kd alone. Despite a similar partial response rate in the two therapy arms (87% vs. 83%), IsaKd demonstrated a greater depth of response with higher rates of very good partial responses (73% vs. 56%) and patients who achieved MRD negativity (sensitivity 10^−5^, 30% vs. 13% *p* < 0.0004) [150].

The Phase 3 CANDOR study compared Kd to Kd plus daratumumab (DKd) in myeloma patients with 1–3 prior lines of therapy. Adding daratumumab to Kd resulted in a statistically significant reduction in the risk of death or progression (median PFS, not reached vs. 16 months; HR 0.63, *p* = 0.0014) when compared with Kd alone; this benefit, to the advantage of patients treated in the DKd arm, was also observed in cases of previous exposure (HR 0.52) or refractoriness to lenalidomide (HR 0.45) [151].

The phase III study CASTOR, the sister trial of POLLUX, investigated the effects of adding daratumumab to the other standard regimen at relapse, i.e., Vd, and compared them (DaraVd vs. Vd). Among patients receiving daratumumab in combination with Vd, a clear advantage was demonstrated over patients in the control arm (Vd) in terms of ORR (84 vs. 63.2%, *p* < 0.001), of CR (23% vs. 10%, *p* = 0.001), and patients with MRD negativity (12% vs. 2%, sensitivity of 10^−5^), which translated into a significantly longer median PFS (median, 16.7 vs. 7.1 months, HR 0.31; *p* < 0.001) [152,153].

Panobinostat is one of several histone deacetylase inhibitors being tested for the treatment of myeloma. In the phase III PANORAMA-1 study in patients with RRMM and 1–3 prior lines of treatment but not bortezomib-refractory, panobinostat was associated with Vd (PanoVd) and compared with Vd. Despite similar ORR in the two treatment arms (60.7% vs. 54.6%; *p* = 0.09), median PFS was significantly greater in patients in the PanoVd arm than in patients in the Vd arm (12.0 vs. 8.1 months; HR, 0.63; *p* < 0.0001).

Despite the PFS benefit, there was no difference in OS between the two arms (median, 33.6 vs. 30.4 months). Adverse events were more common in the PanoVd arm, particularly diarrhea, thrombocytopenia, and asthenia [154].

Pomalidomide is a third generation IMiD, an analog of thalidomide and lenalidomide, that was tested in combination with dexamethasone (PomDex) and compared to dexamethasone alone in highly pretreated RRMM patients (median of five prior lines of therapy). The PomDex combination was shown to induce a significantly higher ORR than Dex alone (21% vs. 3%; *p* < 0.001) and to significantly increase both median PFS (4 vs. 2 months; *p* < 0.001) and median OS (NR vs. 8 months, *p* < 0.001) [155].

The Phase 3 ICARIA study compared the triplet of adding isatuximab to the standard pomalidomide and dexamethasone doublet (IsaPd) in 307 patients treated with at least two prior lines of therapy, including lenalidomide and a proteasome inhibitor. Isa-Pd increased at least the partial response rate (60% vs. 35%) and statistically significantly prolonged median PFS (11.5 vs. 6.5 months; HR 0.596, *p* = 0.001) when compared to the Pd doublet [156]. Importantly, IsaPd demonstrated a reduction in the risk of death or progression when compared with Pd, even in lenalidomide-refractory patients (HR: 0.59).

Further, 559 multiple myeloma patients who had received at least one prior line of therapy were enrolled in the OPTIMISMM study and randomized to receive Pd or Pd plus bortezomib (PVd). Adding bortezomib to Pd increased at least partial response rates (82% vs. 50%) and reduced the risk of death or progression by 39% when compared with the Pd doublet (median PFS, 11 vs. 7 months; HR 0.61, *p* < 0.0001). PVd demonstrated a reduced risk of death or progression when compared with Pd, even in lenalidomide-refractory patients (HR: 0.65) [157].

### 4.4. Therapeutic Options for Patients Refractory to Immunomodulators, Proteasome Inhibitors and Anti-CD38 Monoclonal Antibodies

Belantamab-mafodotin (Belamaf) is an IgG1 monoclonal antibody directed against the B-cell maturation antigen (BCMA) expressed by myeloma cells and conjugated with a direct agent against microtubules, monomethyl auristatin F (MMAF). Belamaf acts both through a direct apoptotic mechanism linked to the transport of MMAF within the plasma cell and through immunological mechanisms such as antibody dependent cellular phagocytosis (ADCP) and antibody dependent cellular cytotoxicity (ADCC). Regulatory approval of Belamaf was gained for patients with relapsed and refractory myeloma to immunomodulators, proteasome inhibitors, and anti-CD38 monoclonal antibodies, based on results from the Phase 2 DREAMM-2 study, in which two doses of belamaf were tested (2.5 and 3.4 mg/kg) in 196 highly pretreated relapsed and refractory MM patients (median of 6–7 prior lines of therapy). About one-third of patients receiving two doses of belamaf achieved at least a partial response, while about one-fifth achieved VGPR. Median PFS was 2.9 months in patients treated with the 2.5 mg/kg dose and 4.9 months in patients treated with the 3.4 mg/kg dose [158]. Based on efficacy and toxicity data, the dose of 2.5 mg/kg administered every 21 days was approved.

The XPO1 molecule is overexpressed in myeloma cells and is capable of transporting tumor suppressors outside the cell nucleus. Selinexor, its inhibitor, was tested in a phase 2 study in patients with triple-refractory MM, i.e., refractory to immunomodulators, proteasome inhibitors, and anti-CD38 monoclonal antibodies. In the Phase 2 study, published by Chari A. et al., selinexor (80 mg twice weekly) in combination with dexamethasone (Sd) was tested in 122 patients with a median of seven prior lines of treatment; 26% of patients who received Sd achieved at least a partial response, while median PFS and OS were 3.7 and 8.6 months [159]. 

One of the parameters for evaluating the efficacy of therapies for MM is the ability of the therapy itself to “cytoreduce” the neoplastic mass. This parameter is based on the detection of the amount of monoclonal protein circulating in the serum and urine, an indirect expression of the amount of neoplastic plasma cells. The identification and quantification of the monoclonal component is performed by immunofixation and protein electrophoresis, respectively [160]. For the evaluation of the response of patients affected by oligosecreting myeloma, the measurement of circulating free light chains (kappa or lambda, serum FLC) has been introduced. In addition to the search for the monoclonal component in serum and urine, investigation of the hematopoietic marrow allows the detection and quantification of monoclonal plasma cells present in the marrow environment.

According to the response criteria published by the International Myeloma Working Group (IMWG) in 2006 [160], the response to treatment is subdivided into five different categories: stable disease (SD), minimal response (MR), partial response (PR), very good partial response (VGPR), and complete response (CR). Disease progression (PD), on the other hand, is defined by the absence of response during treatment or by the recurrence of disease following a previously acquired response.

To correctly define a complete remission of the disease if the serum and/or urine monoclonal component is no longer detectable by electrophoresis and immunofixation, it is necessary to proceed with the quantification of the residual plasma cells at the medullary level. Initially, a quota of residual bone marrow monoclonal plasma cells of less than 5% was required for the definition of CR. Subsequently, the definition of stringent complete response (sCR) was introduced, for which both the total absence of bone marrow monoclonal plasma cells and the concomitant normalization of the ratio between serum free light chains (FLC ratio) are required [38].

The importance of this treatment response categorization derives from the prognostic value inherent in the different response categories. In fact, it has been demonstrated, both in young patients eligible for autologous transplantation and in elderly patients not eligible for high-dose chemotherapy, that the achievement of a complete response is correlated with better PFS and OS [161,162,163,164].

Obtaining CR has thus become one of the goals of multiple myeloma treatment, both in clinical practice and in the context of clinical trials.

The adoption of the autologous stem cell transplant procedure in support of high-dose chemotherapy and the introduction of “new drugs” has significantly increased the rate of complete remissions, stringent or not, to over 50% of patients treated, but problems remain. First of all, most patients will experience a recurrence of myeloma, a sign of the presence, albeit invisible to conventional techniques, of minimal residual disease (MRD) [87,93,165].

Several studies have demonstrated the presence of residual monoclonal plasma cells in the bone marrow environment of patients who had achieved the traditionally defined complete remission, which conventional bone marrow study techniques were unable to detect [166,167]. These studies have also demonstrated that patients in CR, but with positive MRD, had a significantly lower PFS than those of patients in CR and MRD negative, regardless of the method used for the detection of MRD [57,168,169,170].

The techniques used for the study of MRD in myeloma allow simultaneous analysis of hundreds of thousands, up to millions, of bone marrow cells or their DNA, and the detection of any direct or indirect presence of monoclonal plasma cells in the sample examined. These methods are subdivided into cellular (multiparametric flow cytometry, MFC), molecular (allele-specific oligonucleotide-qPCR, ASO-qPCR and next-generation sequencing of VDJ sequences), or imaging (PET/CT) methods.

The evidence generated by MRD studies in myeloma, i.e., the presence of measurable disease with more sensitive techniques than with traditional methods, together with the prognostic value of these results, has led to the need to implement the assessment of response to myeloma treatment with the MRD study.

In August 2016, the IMWG published a review of the response criteria for MM treatment, including the assessment of MRD using the techniques mentioned above [59]. Among those, PET-CT is currently the best tool to assess response after treatment and it is recommended in all patients that need to be evaluated for MRD, thanks to its ability to distinguish active from inactive disease. Indeed, various techniques can be used to detect MRD, including flow cytometry, next-generation sequencing, and imaging modalities such as PET-CT and MRI. PET-CT is currently the most widely used imaging modality for the assessment of MRD in MM patients as it provides a whole-body evaluation of tumor burden and can distinguish active from inactive disease [171,172].

## 5. Current Status and Future Outlook

MM is currently conceived as a non-curable, although treatable, disease. The uncontrolled proliferation of plasma cells in the bone marrow is responsible for end organ damage. Currently, it can be detected thanks to novel clinical and instrumental approaches that can define HR SMM via SLiM-CRAB, MRD assessment, and EMD detection. Nonetheless, owing to its systemic involvement, MM still poses a significant diagnostic challenge for the clinician. Its management focuses on achieving the longest remission period possible, treating eventual relapses and handling any possible complication, i.e., infections, and hyperviscosity. Future approaches will tailor treatments to the patients and specific disease phenotypes thanks to the large number of drugs and therapies developed in the last 15 years, including IMiDs, PIs, monoclonal antibodies (i.e., daratumumab), cell-based therapies, and targeted therapies.

During the diagnostic work-up, the WBLD-CT of the bone is the current standard. (WB-)MRI is discussed for lesions undetectable or difficult detectable via WBLD-CT. PET/CT may arise as a new diagnostic approach, with the highest sensitivity, in the near future. Not alone with 18F-FDG as tracer, but also 13C-MET as a potential MM selective tracer.

Risk stratification is crucial for each NDMM. Some new genetic markers have risen to detect HR status even better in the recent past (e.g., double-hit MM, del(1p), gain1q21). However genetic characterization is always discussed in the scientific community. Walker et al. recently tried an approach to define some SR, some intermediate and some HR patients. For SR MM patients, in TE patients single ASCT is recommended, in TNE patients recommended SOC therapy would be something like Dara-RD (MAIA).

Therapy decision in MM is individual for efficacy and patient safety concerns. For the TE fit MM patient the therapy decision is currently unchallenged Dara-VTD or Dara-VRD. For the TE MM patient with co-morbidities the therapy decision must be highly individual. Patients with renal damage may benefit from VCD or early bortezomib application. When neuropathy is present before therapy begin, Dara-VRD might be a good choice. Regarding TNE MM patients, if in fit or unfit condition, Dara-RD seems to be good choice in first-LOT. Of note, therapy decision is always dependent on individual parameters.

**Figure 3:**
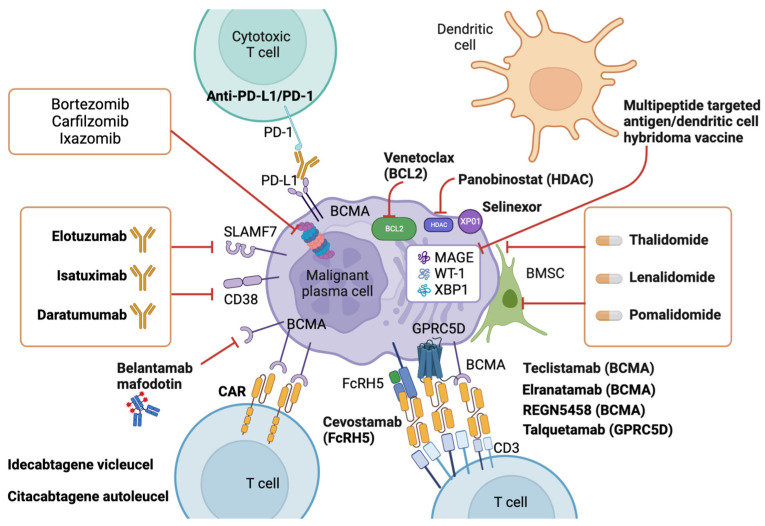
Modern therapy in multiple myeloma. This figure illustrates the pathomechanisms of modern therapeutic approaches for multiple myeloma (MM), which target key pathways involved in MM pathogenesis and progression. The figure highlights the following therapeutic strategies: (i) proteasome inhibitors: Proteasome inhibitors, such as bortezomib and carfilzomib, inhibit the activity of the proteasome complex, leading to the accumulation of misfolded proteins and induction of apoptosis in MM cells. (ii) Immunomodulatory drugs: Immunomodulatory drugs, such as lenalidomide and pomalidomide, modulate the immune microenvironment in MM by inhibiting the production of pro-inflammatory cytokines, enhancing T cell function, and promoting natural killer cell activity. (iii) Monoclonal antibodies: Monoclonal antibodies, such as daratumumab and elotuzumab, target specific antigens on MM cells, leading to their destruction through antibody-dependent cellular cytotoxicity (ADCC) or complement-dependent cytotoxicity (CDC). (iv) Cell-based therapies: Cell-based therapies, such as chimeric antigen receptor (CAR) T cell therapy, involve the engineering of T cells to express CARs that recognize and kill MM cells (v) Targeted therapies: Targeted therapies, such as inhibitors of the phosphoinositide 3-kinase (PI3K)/AKT/mTOR pathway or the B-cell lymphoma-2 (BCL-2), histone deacetylases (HDAC) and Exportin-1 (XPO1) family of proteins, target specific signaling pathways or molecules that are dysregulated in MM cells, leading to their inhibition and apoptosis. MAGE, WT-1, and XBP1 are important targets in myeloma research, offering potential avenues for novel therapies. MAGE (Melanoma-Associated Antigen) and WT-1 (Wilms Tumor 1) are cancer-testis antigens often overexpressed in multiple myeloma, making them attractive targets for immunotherapies like cancer vaccines and adoptive T-cell therapies. Targeting these antigens aims to induce an immune response specifically against myeloma cells, sparing healthy tissues. Additionally, XBP1 (X-Box Binding Protein 1) is a transcription factor critical for plasma cell differentiation and survival. Inhibiting XBP1 holds promise as a therapeutic strategy to disrupt the survival mechanisms of myeloma cells, potentially leading to improved treatment outcomes. Research focusing on these targets shows great potential in advancing precision medicine approaches for multiple myeloma. Finally, novel immunological targeting strategies are represented. Teclistamab: a promising therapy that targets B-cell maturation antigen (BCMA), a cell surface protein highly expressed on multiple myeloma cells. Teclistamab is designed to direct the immune system to attack BCMA-expressing myeloma cells. Elranatamab: an investigational therapy also targeting BCMA, aiming to trigger the immune system to eliminate myeloma cells expressing this antigen. Elranatamab holds potential as a novel treatment for multiple myeloma. REGN5458: another BCMA-targeting therapy that seeks to harness the immune system to target and destroy BCMA-expressing myeloma cells. REGN5458 represents an exciting advancement in the field of multiple myeloma treatment. Talquetamab: an innovative therapy that targets G protein-coupled receptor family C group 5 member D (GPRC5D), a protein found on the surface of myeloma cells. Talquetamab aims to engage the immune system in attacking GPRC5D-expressing myeloma cells. Cevostamab: a potential therapeutic option that targets Fc receptor homolog 5 (FcRH5), a cell surface protein expressed on myeloma cells. Cevostamab aims to induce an immune response against FcRH5-expressing myeloma cells. Idecabtagene vicleucel: an innovative approach using chimeric antigen receptor (CAR) T-cell therapy, specifically Idecabtagene vicleucel, to target and eliminate multiple myeloma cells. This personalized treatment involves modifying patients’ own T-cells to express a CAR that recognizes and attacks myeloma cells. Citacabtagene autoleucel (Cita-cel) is another chimeric antigen receptor (CAR) T-cell therapy used in the treatment of multiple myeloma. It involves engineering a patient’s T-cells to express a CAR that targets BCMA. Understanding the patho-biological mechanism of modern therapeutic approaches for MM is crucial for the development of effective treatment strategies alone and in combination with already approved agents that can improve patient outcomes. Combination therapies that target multiple pathways and mechanisms may offer the best chance for achieving durable responses and long-term disease control in MM [30]. More details are provided in the text. Created by BioRender, publication license n. CW25N085PL.

The development of novel therapeutics over the past two decades has significantly improved patient outcomes, with a focus on diverse mechanisms of action against the plasma cell clone, protein degradation pathways, and monoclonal antibodies targeting surface antigens. PD-1 (Programmed Cell Death Protein 1) and PD-L1 (Programmed Death-Ligand 1) are crucial components of the immune system’s checkpoint pathway, playing a significant role in regulating immune responses. In the context of multiple myeloma, these molecules have garnered attention due to their potential as therapeutic targets. In multiple myeloma, cancer cells can exploit the PD-1/PD-L1 pathway to evade the immune system’s attack. PD-L1, often expressed on the surface of myeloma cells, interacts with PD-1 receptors on immune cells like T cells, leading to immune suppression and allowing the tumor to escape destruction. Researchers have been investigating the use of PD-1 and PD-L1 inhibitors in multiple myeloma treatment. These inhibitors block the interaction between PD-L1 and PD-1, thus reactivating the immune system and enhancing its ability to recognize and attack myeloma cells. Early clinical trials and studies have shown promising results, with some patients responding positively to PD-1/PD-L1 blockade therapies. However, the use of these inhibitors in myeloma is still in the research and development phase, and more extensive studies are required to determine their long-term effectiveness and safety in treating the disease [173]. As medical research progresses, targeting the PD-1/PD-L1 pathway in multiple myeloma may become a valuable addition to the therapeutic arsenal, potentially improving patient outcomes and quality of life, despite potential side effects [174,175]. These advancements have led to improved response to treatment, remission duration, quality of life, and overall survival rates for MM patients. Although MM remains an incurable disease, the progress made in recent years provides hope for continued advancements in the field and ultimately a cure for this devastating disease with modern therapeutic approaches (Figure 3).

## Data Availability

Data can be required to the corresponding authors.
